# The negative association between the docosapentaenoic acid intake and the incidence of AMD based on NHANES 2005–2008

**DOI:** 10.3389/fnut.2024.1435775

**Published:** 2024-07-25

**Authors:** Baiwei Xu, Yi Hu, Jie Di, Zhongwei Liu, Ziyan Yu, Lin Han, Yuan Ning

**Affiliations:** ^1^Department of Ophthalmology, The Fourth Affiliated Hospital of China Medical University, Shenyang, China; ^2^Eye Hospital of China Medical University, Shenyang, China; ^3^Key Lens Research Laboratory of Liaoning Province, Shenyang, China; ^4^Department of Anesthesiology, Shengjing Hospital of China Medical University, Shenyang, China

**Keywords:** age-related macular degeneration, cross-sectional study, national health and nutrition examination survey, fatty acid, docosapentaenoic acid

## Abstract

**Introduction:**

Age-related macular degeneration (AMD) is an ophthalmic disease that causes visual impairment and is one of the leading causes of blindness in the elderly. Fatty acids are essential nutrients required by the body and play a cornerstone role in the life activities of the body. Many studies have reported that fatty acids are involved in the development of AMD. To confirm this association, we conducted the present study.

**Methods:**

We analyzed the association between all fatty acid intake and AMD using National Health and Nutrition Examination Survey (NHANES) data from 2005–2008. Quantile regression was performed to assess the effect of fatty acids on AMD at different intake levels.

**Results:**

After adjusting for covariates, only saturated fatty acids showed no significant difference between AMD patients and non-AMD patients (23.64 g vs. 26.03 g, *p* = 0.052). Total fat (70.88 g vs. 78.86 g, *p* = 0.024), monounsaturated fatty acids (25.87 g vs. 28.95 g, *p* = 0.019), polyunsaturated fatty acids (15.10 g vs. 17.07 g, *p* = 0.017) showed significant differences between the two groups. When AMD was considered as an outcome, the association between AMD and docosaentaenoic acid (DPA) was negative in the multivariate logic model (model 1: OR = <0.001, 95% CI = <0.001 ~ 0.734; model 2: OR = <0.001, 95% CI = <0.001 ~ 0.002; model 3: OR = <0.001, 95% CI = <0.001 ~ 0.002). In the quantile regression, DPA was shown to be negatively associated with the presence of AMD only in the fourth quartile in model 2 and model 3 (model 2: OR = <0.001, 95% CI = <0.001 ~ 0.927; model 3: OR = <0.001, 95% CI = <0.001 ~ 0.775).

**Discussion:**

Therefore, based on above results, we concluded that DPA intake could prevent the development of AMD.

## 1 Introduction

Age-related macular degeneration (AMD) is one of the leading causes of blindness in the elderly. And globally, it is predicted that there will be 196 million adults with AMD in 2020, increasing to 288 million by 2040 (1, 2). It is a multifactorial disorder and strong association were found between age and smoking and the disease progression, while other factors including diet, genetic predisposing factors, and other environmental risk factors may also affect it (3–5). AMD is progressive blinding retinopathy, and its development is mainly related to retinal pigment epithelium, choroidal neovascularization, Bruch’s membrane thickening, and Drusen formation (6, 7). Drusen is an extracellular sediment located between Bruch’s membrane and the retinal pigment epithelium. Its contents include lipids, lipoproteins, and oxidized lipids, which are the main hallmark of AMD (8, 9). Early AMD is characterized by moderate Drusen (63–125 μm) or pigment variants (10), whereas midstage AMD is characterized by larger Drusen (>125 μm) and pigment abnormalities, which may progress to late-stage geographic atrophy and choroidal neovascularization, atrophic or exudative/neovascular AMD (11, 12).

Fatty acids, as modulators of cell membrane characteristics and energy supply to cells, are an important nutrient for maintaining normal physiological functions. Fatty acid derivatives can mediate cell signaling (13–15), so fatty acids are an important part of the outer segment of retinal photoreceptors (16, 17), and interact with rhodopsin, which also plays a role in light transmission (18–21). Docosahexaenoic acid (DHA) is a polyunsaturated fatty acid widely found in the retina and brain (22). It exists in the disk membrane of the outer segment of retinal rod photoreceptors and is essential for retinal function (23), and can prevent damage caused by strong light and oxidative stress (24).

Previous studies have found that high dietary intake of trans fatty acid may contribute to the development of AMD, and higher intake of monounsaturated fatty acids and polyunsaturated fatty acids was inversely proportional to the development of AMD, particularly in the study by Jiang et al. in 2021 showed that greater dietary omega-3 polyunsaturated fatty acids intake may contribute to a lower chance of developing AMD (25). Consumption of foods rich in omega-3 polyunsaturated fatty acids has been identified as a potential new dietary approach for AMD prevention (26). In 2022, a study on the risk of early AMD in the Japanese population also showed that adequate intake of dietary fatty acids is needed to maintain retinal homeostasis and prevent AMD (27). Preventing and delaying the emergence or progression of AMD can be achieved by adding some types of fatty acids and lipid derivatives to the daily diet (28). Multiple retrospectives and prospective epidemiological studies have also shown that dietary rich in long-chain polyunsaturated fatty acids contributes to a lower incidence of AMD (29), and dietary low intake of (N-3) long-chain polyunsaturated fatty acids is associated with a higher risk of macular degeneration (30). In the Age-Related Eye Diseases Study (AREDS), a large prospective study that looked at factors for progression to advanced AMD, subjects with the lowest intakes were 50% less likely to develop AMD than those with the highest intakes of foods rich in long-chain unsaturated fatty acids, Subsequent studies have also supplemented the efficacy of primary polyunsaturated fatty acids (PUFA) in preventing progression to advanced AMD or wet AMD (31).

However, there are relatively few published articles assessing the relationship between fatty acid intake and AMD. Understanding whether fatty acid influence the development and progression of AMD may help prevent AMD and provide opportunities for interventions to treat the disease. The primary objective of this study was to examine the potential connection between intake of multiple dietary fatty acids and the development of AMD using statistical analysis of NHANES data.

## 2 Materials and methods

### 2.1 Data source and subjects selection

This study is a cross-sectional study based on data from National Health and Nutrition Examination Survey (NHANES) 2005–2008, including 4,996 subjects. NHANES is a large nationwide cross-sectional study performed by the National Center for Health Statistics (NCHS). NHANES subjects were all U.S. masses randomly selected based on a sampling design, who underwent universal examination and signed informed consent. The NCHS research ethics review board approved the survey protocol for NHANES (32).

### 2.2 Defining criteria for AMD

Participants aged 40 years or older were taken binocular non mydriatic fundus photographs in Mobile Examination Center (MEC) using the Canon Non-Mydriatic Retinal Camera CR6-45NM. Digital images were copied onto Digital Versatile Disk (DVD) and shipped to the University of Wisconsin and assessed by a rater at the University of Wisconsin. Digital images were evaluated and classified into 3 severity levels, no AMD, early AMD, late AMD. Early AMD is defined as the presence or absence of Drusen and/or pigmentary abnormalities; late AMD is caused by exudative arm signs and/or geographic atrophy (33). In this study, patients who were rated as having early AMD or late AMD in at least one eye were defined as having AMD.

### 2.3 Determination of intake of fatty acids and daily energy intake

Dietary data were collected in the in-person interview using the Automated Multiple Pass Method (AMPM). The AMPM is a United States Department of Agriculture’s dietary data collection instrument and a fully computerized recall method. The NHANES MEC provided a set of measuring guides that facilitated participants to describe the amount of foods they had ingested (34, 35).

NHANES performed dietary data statistics for two consecutive days, and we considered the mean of two daily dietary data for each subject as the final dietary intake data in an effort to obtain an outcome that more closely approximated the true level of life. All available dietary fatty acid data included in NHANES were included in this study.

### 2.4 Covariates assessment

Socio-demographic variables including age, race/ethnicity, gender, and educational level were obtained by computer-assisted in-person interview (36). Serum cholesterol, serum triglycerides, serum low density lipoprotein, cataract surgery, smoking were also included as covariates. Smoking status was defined by serum cotinine levels to reflect both direct and indirect smoking quantity.

### 2.5 Statistical analysis

All statistical analyses were performed using SAS 9.4 and R software 4.1.3. NHANES uses a stratified, multistage sampling method, so we incorporated sampling weights and strata, sampling units in our statistical analysis to account for the complex sampling design. Continuous variables were presented with mean and standard error (SE), and categorical variables were presented with percentages and SE, and the Chi-square tests or T-test was used to compare patients’ demographic characteristics. Logistic regression models were used to determine the association of various trace element intakes with the presence of cataracts. Model 1 was adjusted by age, race, gender, educational level, daily energy intake. Model 2 = Model 1 and adjusted by Serum cholesterol, serum triglycerides, serum low density lipoprotein. Model 3 = Model 2 and adjusted by serum cotinine, cataract surgery. Since a significant association between docosapentaenoic acid and AMD was observed, we further performed quantile regression between Docosaentaenoic acid (DPA) and AMD.

## 3 Results

### 3.1 Description of baseline information of the study sample

**Figure 1:**
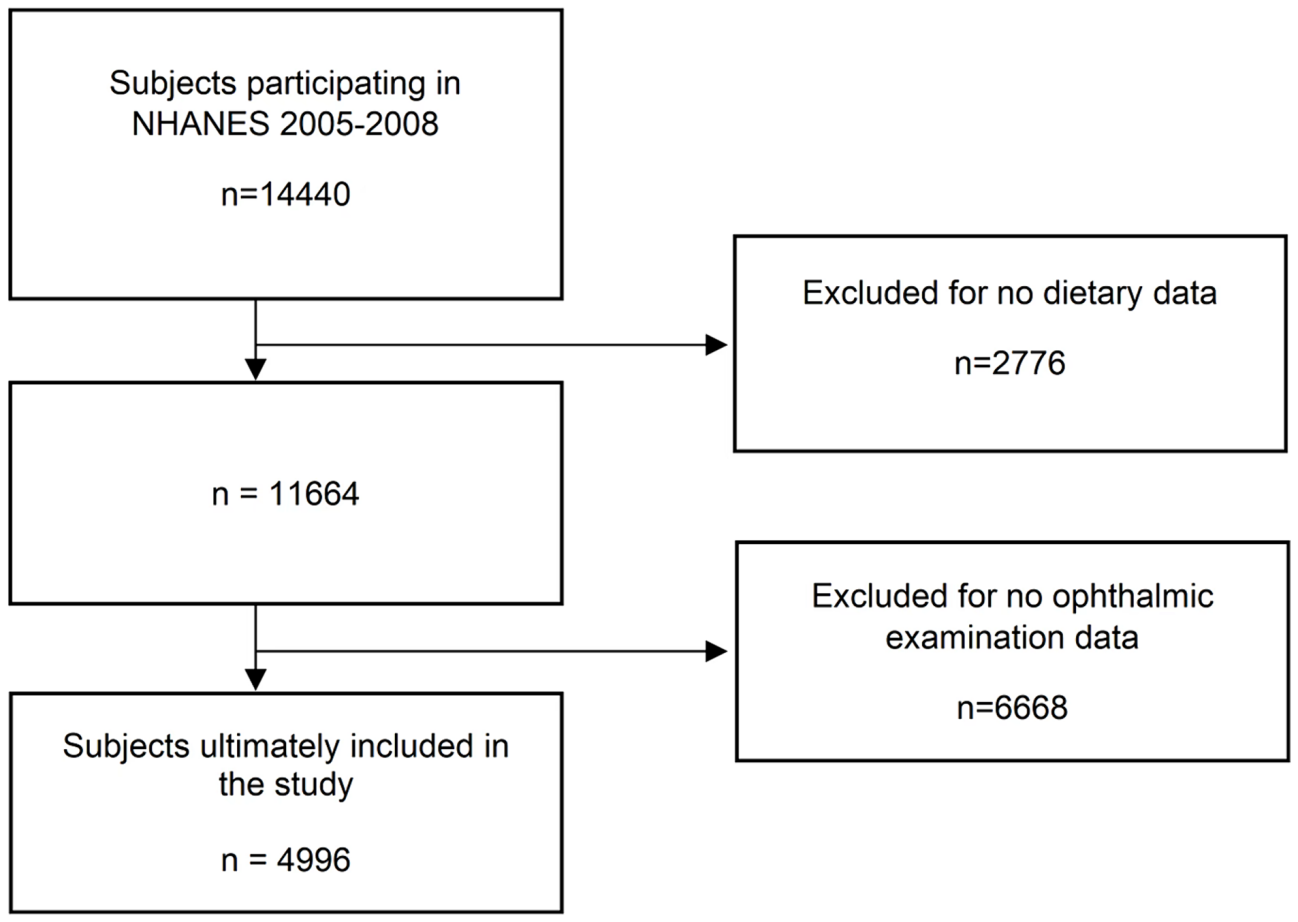
Screening process.

Based on the study design of NHANES, we selected a total of 20,497 subjects for inclusion in this study. After screening, 4,996 subjects were finally included, and the remaining 15,501 subjects were excluded due to missing dietary data or ophthalmological examination data. Figure 1 demonstrates the screening flow.

Table 1 shows the demographic data as well as other characteristic data of the participants with and without AMD. The number of subjects with AMD comprised 6.7% of the subject population after weighting. Older age (68.78 years vs. 55.85 years, *p* < 0.001) and non-Hispanic white individuals (88.40% vs. 77.40%, *p* < 0.001) were more likely to have AMD. Gender and education level had no significant effect on AMD.

**Table 1:** Baseline information for the study sample.

Variables	AMD status		*p*-value
	AMD (+)	AMD (−)	
**Continuous variables, mean (SE)**			
Age (years)	68.78 (0.88)	55.85 (0.37)	<0.001
**Category variables, % (SE)**			
AMD	6.70 (0.60)	93.30 (0.60)	
Gender			0.44
Male	43.20 (3.00)	45.70 (0.90)	
Female	56.80 (3.00)	54.30 (0.90)	
Race			<0.001
Mexican American	4.10 (1.00)	5.40 (0.70)	
Other hispanic	2.30 (0.80)	3.10 (0.60)	
Non-hispanic white	88.40 (1.70)	77.40 (2.10)	
Non-hispanic black	3.60 (0.70)	9.90 (1.30)	
Other race–including multi-racial	1.60 (0.90)	4.20 (0.70)	
Education level			0.14
Less than 9th grade	10.10 (3.10)	6.10 (0.70)	
9-11th grade (Includes 12th grade with no diploma)	11.70 (2.30)	10.40 (0.80)	
High school grad/GED or equivalent	26.40 (3.10)	26.50 (1.20)	
Some college or AA degree	30.20 (3.60)	27.90 (1.10)	
College graduate or above	21.60 (3.70)	29.00 (1.80)	

### 3.2 Description of daily dietary fatty acid intake and clinical characteristics of the study sample

Table 2 shows the comparison of fatty acid intake and clinical characteristics between AMD and non-AMD groups. Fatty acid intake was significantly higher in the non AMD group than in the AMD group (70.88 g vs. 78.86 g, *p* = 0.024). Saturated fatty acids were not significantly different between the two groups (*p* = 0.052). The AMD group consumed significantly less monounsaturated fatty acids (25.87 g vs. 28.95 g, *p* = 0.019) and polyunsaturated fatty acids (15.10 g vs. 17.07 g, *p* = 0.017) than the non-AMD group. Most monounsaturated fatty acids and polyunsaturated fatty acids also show the above results. Among the clinical characteristics, only daily caloric intake (1816.68 kcal vs. 2033.58 kcal, *p* = 0.001) and history of cataract surgery (31.80% vs. 7.50%, *p* < 0.001) showed a significant association with the onset of AMD.

**Table 2:** Daily dietary fatty acid intake and clinical characteristics of participants with and without AMD.

Variables	AMD (+)	AMD (−)	*p*-value
**Continuous variables, mean (SE)**			
Total fat (g)	70.88 (3.56)	78.86 (0.93)	0.024
Total saturated fatty acids (g)	23.64 (1.84)	26.03 (0.34)	0.052
Total monounsaturated fatty acids (g)	25.87 (1.24)	28.95 (0.35)	0.019
Total polyunsaturated fatty acids (g)	15.10 (0.78)	17.07 (0.22)	0.017
Hexadecenoic acid (g)	1.04 (0.054)	1.20 (0.015)	0.007
Octadecenoic acid (g)	24.22 (1.17)	27.04 (0.33)	0.023
Eicosenoic acid (g)	0.22 (0.015)	0.24 (0.0047)	0.125
Docosenoic acid (g)	0.035 (0.0068)	0.040 (0.0022)	0.37
Octadecadienoic acid (g)	13.41 (0.72)	15.066 (0.20)	0.028
Octadecatrienoic acid (g)	1.35 (0.066)	1.50 (0.021)	0.033
Octadecatetraenoic acid (g)	0.011 (0.0014)	0.016 (0.00099)	0.001
Eicosatetraenoic acid (g)	0.11 (0.0046)	0.14 (0.0023)	<0.001
Eicosapentaenoic acid (g)	0.028 (0.0038)	0.053 (0.0042)	<0.001
Docosapentaenoic acid (g)	0.011 (0.00086)	0.020 (0.0010)	<0.001
Docosahexaenoic acid (g)	0.053 (0.0049)	0.097 (0.0066)	<0.001
Daily energy intake (kcal)	1816.68 (59.64)	2033.58 (20.07)	0.001
Serum cholesterol (mg/dL)	201.30 (3.53)	204.92 (0.92)	0.312
Serum triglycerides (mg/dL)	138.82 (7.26)	149.29 (3.12)	0.16
Low density lipoprotein (mg/dL)	119.42 (4.18)	120.42 (1.01)	0.814
Serum cotinine (ng/dL)	59.92 (8.64)	61.97 (3.92)	0.814
**Category Variables, % (SE)**			
Cataract surgery			<0.001
(+)	31.80 (3.00)	7.50 (0.50)	
(−)	68.20 (2.80)	92.50 (1.10)	

### 3.3 Association between daily dietary fatty acid intake and the presence of AMD

Table 3 shows the associations that existed between the intake of daily dietary fatty acid intake and AMD as addressed by multivariate logistic regression models. A significant negative association between DPA intake and incident AMD was shown in all models (model 1: OR = <0.001, 95% CI = <0.001 ~ 0.734; model 2: OR = <0.001, 95% CI = <0.001 ~ 0.002; model 3: OR = <0.001, 95% CI = <0.001 ~ 0.002). No significant association with AMD was observed for the intakes of other fatty acid.

**Table 3:** Association between intake of daily dietary fatty acid intake and AMD.

Variables	Model 1^a^ OR (95% CI)	*p*-value	Model 2^b^ OR (95% CI)	*p*-value	Model 3^c^ OR (95% CI)	*p*-value
Hexadecenoic acid	1.120 (0.711 ~ 1.764)	0.616	0.856 (0.503 ~ 1.456)	0.554	0.905 (0.545 ~ 1.503)	0.690
Octadecenoic acid	1.000 (0.963 ~ 1.038)	0.291	1.0180 (0.954 ~ 1.087)	0.578	1.013 (0.950 ~ 1.079)	0.685
Octadecadienoic acid	1.0270 (0.977 ~ 1.080)	0.291	0.989 (0.899 ~ 1.089)	0.823	0.999 (0.910 ~ 1.096)	0.980
Octadecatrienoic acid	0.835 (0.606 ~ 1.149)	0.258	1.038 (0.647 ~ 1.667)	0.872	1.0130 (0.633 ~ 1.620)	0.956
Octadecatetraenoic acid	254.678 (0.405~ > 999.999)	0.0890	>999.999 (0.751~ > 999.999)	0.0561	>999.999 (0.242~ > 999.999)	0.0831
Eicosatetraenoic acid	0.742 (0.0720 ~ 7.603)	0.795	2.855 (0.047 ~ 174.272)	0.606	2.573 (0.052 ~ 127.681)	0.625
Eicosapentaenoic acid	9.401 (0.131 ~ 676.532)	0.293	0.112(<0.001 ~ 40.369)	0.454	0.066 (<0.001 ~ 21.264)	0.344
Docosapentaenoic acid	<0.001 (<0.001 ~ 0.734)	0.0440	<0.001 (<0.001 ~ 0.002)	0.00540	<0.001 (<0.001 ~ 0.002)	0.00590
Docosahexaenoic acid	0.0860 (~0.005 ~ 1.551)	0.0940	1.0530 (0.0480 ~ 23.0310)	0.973	1.420 (0.071 ~ 28.556)	0.813

^a^Model 1: adjusted for age, race, gender, educational level, daily energy intake. ^b^Model 2: further adjusted for serum cholesterol, serum triglycerides, serum low density lipoprotein. ^c^Model 3: further adjusted for serum cotinine, cataract surgery.

### 3.4 Relationship of different quartiles of DPA with the presence of AMD

Table 4 demonstrates the analysis of the association of different grades of DPA intake with AMD after dividing DPA intake into quartiles. Significant negative correlations between the fourth quartiles and the prevalence of AMD were seen in model 2 and model 3 (model 2: OR = <0.001, 95% CI = <0.001 ~ 0.927; model 3: OR = <0.001, 95% CI = <0.001 ~ 0.775).

**Table 4:** Association between DPA intake levels and AMD in different quartiles.

Variables		Model 1^a^ OR (95% CI)	*p*-value	Model 2^b^ OR (95% CI)	*p*-value	Model 3^c^ OR (95% CI)	*p*-value
Docosapentaenoic acid intake	Q1	<0.001 (<0.001~ > 999.999)	0.565	<0.001 (<0.001~ > 999.999)	0.750	<0.001 (<0.001~ > 999.999)	0.734
	Q2	>999.999 (<0.001~ > 999.999)	0.590	8.156 (<0.001~ > 999.999)	0.979	17.0250 (<0.001~ > 999.999)	0.983
	Q3	<0.001 (<0.001~ > 999.999)	0.540	<0.001 (<0.001~ > 999.999)	0.525	<0.001 (<0.001~ > 999.999)	0.285
	Q4	<0.001 (<0.001 ~ 1.838)	0.0612	<0.001 (<0.001 ~ 0.927)	0.0495	<0.001 (<0.001 ~ 0.775)	0.0483

^a^Model 1: adjusted for age, race, gender, educational level, daily energy intake. ^b^Model 2: further adjusted for serum cholesterol, serum triglycerides, serum low density lipoprotein. ^c^Model 3: further adjusted for serum cotinine, cataract surgery.

## 4 Discussion

Data from NHANES 2005–2008 served as the foundation for our cross-sectional investigations on the relationship between AMD and fatty acid intake. First, we compared fatty acid intake between AMD and non-AMD groups and found significant differences in most fatty acids except saturated fatty acids. A multivariable logistic regression analysis was subsequently performed, and only DPA was found to be significantly negatively related the development of AMD. In subsequent quantile regression, significant negative associations of AMD and DPA in all models were observed only in the fourth quartile. The above results suggest that high-dose intake of DPA is helpful in preventing the occurrence of AMD.

DPA is commonly considered as a secondary ω-3 Fatty acids (n-3 FA) are often ignored in the research (37). Due to the limited availability of pure compounds, it has not been widely studied. In fact, it is an intermediate product of DHA and eicosapentaenoic acid (EPA) metabolism (38). DHA, EPA, and DPA are the three PUFA generated via a process of desaturation and elongation of alphalinolenic acid (ALA) (39). The process of converting DPA into DHA is more tortuous than the direct conversion of EPA to DPA (40), including chain elongation, desaturation, and transfer to peroxisomes shortens the chain to form DHA (41).

There has been many prior study on the impacts and relationships between n-3 FA and a variety of clinical manifestations; the majority of this work, however, concentrated on EPA and DHA as the active ingredients (39). At present, there are few clinical studies on the association between pure DPA and AMD, though DPA has many similarities with EPA and DHA in function and metabolism *in vivo*. The relationship between n-3 FA and AMD has been extensively studied. However, all the shreds of evidence about EPA, DHA and AMD are inconsistent (42–54). Our finding broadly supports the work of other studies in this area linking n-3 FA with delaying or preventing AMD. These results are similar to those reported by observational studies, clinical trial, meta-analyses and cross-sectional studies. Many observational studies have analyzed the correlation between fish and the progression of AMD. The most recent results of these investigations demonstrate a detrimental relationship between fish consumption and AMD (43–47). At the same time, many clinical experiments have confirmed that DHA and EPA, two n-3 FA rich in fish, may be able to halt or prevent the progression of AMD (43, 50). One of the studies measured the relationship between DHA, EPA and AMD in plasma, giving more credible evidence than n-3 FA mount of intake (55).

However, a systematic analysis of clinical research involving EPA, DHA, and placebo groups revealed no connection between PUFA intake and AMD progression over the course of 5 years (56). And a large multicenter, randomized, double masked, placebo-controlled phase 3 study. The Age Related Eye Disease Study 2 (AREDS2) also reached the same conclusion. In AREDS2, the intake dose of participants is DHA (350 mg) + EPA (650 mg) (54). However, considering that the nutrition supplement of the placebo group in AREDS2 is in AREDS to confirm its negative cor-relation with AMD, we still cannot have a real placebo group data for comparison (57). Another earlier randomized prospective study, Nutritional AMD Treatment 2 Study (NAT2), although small in scale, had a real placebo group as a control (57). The data of NAT2 showed that the incidence of choroidal neovascularization (CNV) in patients with unilateral exudative AMD (unilateral exudative AMD) with high plasma concentrations of DHA and EPA decreased significantly for three consecutive years. The results suggested that higher concentrations of DHA and EPA in plasma had significant effects on AMD (53). Although AREDS2 and other studies came to the conclusion that n-3 FA has no discernible effect on AMD, new research opposes this conclusion. We speculate that there are two reasons for this disagreement. First, the nutrition supplement settings of the placebo group have included effective ingredients for AMD (58), which makes it difficult to highlight the role of n-3 FA in statistical data. Secondly, Omega-3 PUFA is a complex family. At present, all research results on fish intake and AMD development are negatively correlated, but the results of DHA and EPA supplementation alone are contradictory. Maybe other Omega-3 PUFAs contained in fish also have a role in AMD development (51).

The current research has provided some possible mechanisms for why n-3 FA is negatively correlated with AMD. First, current research has shown that the pathogenesis of AMD is closely related to oxidative stress, inflammatory reaction (59) and CNV (60). n-3 FA might be deeply engaged in the aforementioned causes. DHA is the highest content of polyunsaturated fatty acid in photoreceptor membranes (61), which is retained in photoreceptor cells to prevent peroxidation (62, 63). If the concentration of DHA in the plasma is low, the fragments formed by its oxidation accumulate in the eyes (64), and the hapten produced by the oxidative cleavage of DHA will induce immune reaction, produce inflammation, and finally lead to the occurrence of AMD. In addition, n-3 FA can also metabolize various anti-inflammatory factors to regulate immune response (65). Current studies have confirmed that n-3 FA can inhibit CNV (48), which is the primary reason of the final blindness in exudative AMD. It has been shown that n-3 FA derivatives can inhibit CNV by inhibiting the angiogenesis of endothelial cells (66), but do not affect vascular endothelial growth factor (VEGF) (67). In addition, the latest research on DPA showed that DPA pretreatment of bovine aortic endothelial cells inhibited their migration activity due to the stimulation of VEGF, the same pretreatment inhibited angiogenesis (68), which indicated that DPA was a angiogenesis inhibitor. The above research provides some clues for the mechanism of DPA’s function on AMD, allowing us to continue to explore in depth along with the existing achievements.

Meanwhile, in addition to DPA as n-3 FA, some DPA also participates in metabolic activities as the n-6 fatty acids (n-6 FA), which is the final product obtained from the conversion of linoleic acid (69). So far, no researchers have conducted direct research on the relationship between n-6-DPA and AMD. However, Chen et al.’s study has shown that increasing consumption of n-6 DPA can significantly reduce plasma total cholesterol (70). Meanwhile, excessive deposition of cholesterol in the retina can damage retinal pigment epithelial cells and photoreceptor cells, leading to the formation of vitreous warts and ultimately inducing AMD (71–73). Therefore, we speculate that n-6 DPA has a protective effect on AMD through this cholesterol lowering effect. Meanwhile, several studies have shown that statin drugs have a protective effect on AMD, providing lateral evidence for our viewpoint (74, 75). Unfortunately, due to limitations in NHANES raw data, we were unable to distinguish the form of DPA in this study. However, in summary, both n-3 DPA and n-6 DPA have played beneficial roles in preventing AMD.

Our study has certain weaknesses. For example, fatty acid intake data are derived from the subject’s verbal recall, which is subject to error with actual intake; cross sectional studies alone cannot prove causality among study subjects et al. These errors were derived from the study design of NHANES itself. Even so, our study’s large sample size and representative sampling strategy do result in fresh, trustworthy data supporting the link between fatty acid intake and AMD at the level of overall findings.

## 5 Conclusion

The intake of fatty acids has a strong association with the occurrence of AMD. High dose intake of docosapentaenoic acid may help prevent the development of AMD.

## Data availability statement

The original contributions presented in the study are included in the article/supplementary material, further inquiries can be directed to the corresponding authors.

## Author contributions

BX: Data curation, Formal analysis, Methodology, Software, Writing – original draft, Writing – review & editing. YH: Conceptualization, Writing – original draft, Writing – review & editing. JD: Writing – original draft, Writing – review & editing. ZL: Formal analysis, Investigation, Methodology, Software, Writing – original draft. ZY: Validation, Writing – review & editing. LH: Funding acquisition, Project administration, Supervision, Validation, Writing – review & editing. YN: Investigation, Project administration, Supervision, Validation, Writing – review & editing.
